# Narcissistic personality traits and prefrontal brain structure

**DOI:** 10.1038/s41598-021-94920-z

**Published:** 2021-08-03

**Authors:** Igor Nenadić, Carsten Lorenz, Christian Gaser

**Affiliations:** 1 Department of Psychiatry and Psychotherapy, Philipps Universität Marburg, Rudolf-Bultmann-Str. 8, 35039 Marburg, Germany; 2 Department of Psychology, Goethe-Universität Frankfurt, Frankfurt, Germany; 3 Department of Psychiatry and Psychotherapy, Jena University Hospital, Jena, Germany; 4 Department of Neurology, Jena University Hospital, Jena, Germany

**Keywords:** Social neuroscience, Brain imaging, Human behaviour

## Abstract

Narcissistic traits have been linked to structural and functional brain networks, including the insular cortex, however, with inconsistent findings. In this study, we tested the hypothesis that subclinical narcissism is associated with variations in regional brain volumes in insular and prefrontal areas. We studied 103 clinically healthy subjects, who were assessed for narcissistic traits using the Narcissistic Personality Inventory (NPI, 40-item version) and received high-resolution structural magnetic resonance imaging. Voxel-based morphometry was used to analyse MRI scans and multiple regression models were used for statistical analysis, with threshold-free cluster enhancement (TFCE). We found significant (*p* < 0.05, family-wise error FWE corrected) positive correlations of NPI scores with grey matter in multiple prefrontal cortical areas (including the medial and ventromedial, anterior/rostral dorsolateral prefrontal and orbitofrontal cortices, subgenual and mid-anterior cingulate cortices, insula, and bilateral caudate nuclei). We did not observe reliable links to particular facets of NPI-narcissism. Our findings provide novel evidence for an association of narcissistic traits with variations in prefrontal and insular brain structure, which also overlap with previous functional studies of narcissism-related phenotypes including self-enhancement and social dominance. However, further studies are needed to clarify differential associations to entitlement vs. vulnerable facets of narcissism.

Narcissism refers to a set of personality traits incorporating cognitive, emotional, and behavioural features, which are commonly conceptualised around facets of grandiosity, entitlement, and vulnerability^1–3^. Current conceptualisations of narcissism therefore consider a bipolarity of grandiose vs. vulnerable narcissism or multipolarity of major facets, often evolving around deficits in maintaining functional levels of self-esteem, with such traits being common in the general population and not uniformly linked to dysfunction or distress^2^.

Narcissistic traits have been studied both in social or personality psychology as well as clinical contexts, especially with reference to narcissistic personality disorder (NaPD)^4–6^. While the case has been made that clinical research on narcissistic personality disorder might benefit from data obtained in non-clinical studies of narcissistic traits^7^, the relation between the conceptualisations in these two different lines of research is by no means clear and a matter of ongoing debates and research^3,6,8^. In the subclinical range, narcissistic traits can be associated with positive effects in initial group formation and leadership, but often lead to adverse interactional outcomes over time^9,10^.

Psychometric characterisation of the narcissistic phenotype in general population cohorts has relied on well-established and validated questionnaires, in particular the Narcissistic Personality Inventory (NPI) by Raskin and Hall^11^, which considers aspects of grandiosity, as well as leadership and entitlement^12–16^. Hence, while alternative more recent scales have become available^16–18^, the NPI still remains a widely used instrument^19,20^ with a large database of studies^21,22^.

Given the relevance of narcissism in both clinical and non-clinical research fields, there is an astonishing paucity of neuroscience research relating narcissistic traits or behaviours to either brain function or structure. A pioneering explorative functional magnetic resonance imaging (fMRI) study comparing 11 high-narcissistic vs. 11 low-narcissistic subjects using an empathy paradigm implied decreased deactivation in the right anterior insula in the high-narcissism group^23^, an area implicated in cognitive empathy^24–26^, which can be considered a main factor in developing prosocial behaviours^27,28^. Further functional studies have found correlations of narcissistic traits in clinically healthy subjects in anterior insula and dorsal anterior cingulate and subgenual cingulate cortices during tasks involving social rejection^29^, as well as elevated dorsal anterior cingulate cortex (dACC) response to social rejection stimuli^30^ and self-related visual stimulus processing^31^. In an EEG study, feed-back related negativity in midline frontal areas in an EEG study did not differ between low vs high narcissistic subjects, but a difference in centro-parietal P3 emerged^32^. Together with studies implicating impaired structural white matter connectivity in frontostriatal tracts^33^, this gives rise to (anterior) insula and prefrontal (esp. dACC) involvement in narcissistic behaviours.

In contrast to these cues from functional imaging studies, there is no clear evidence on the brain structural underpinnings, esp. for grey matter. One previous study using cortical thickness measurements reported a negative correlation of PNI (pathological narcissism inventory) scores with right dorsolateral and inferior prefrontal thickness, and cortical volumes in the left medial prefrontal and right dorsolateral prefrontal cortices^34^, while another showed an interaction of gender and NPI scores in the right superior parietal cortex using voxel-based morphometry^35^.

The present study was conducted to test the association of brain structure and narcissistic traits in a non-clinical cohort. In particular, we tested the hypothesis that subclinical narcissistic traits (assessed with the NPI) would be correlated with prefrontal brain structures (as implicated in functional studies and one of the preceding cortical mapping studies) as well as the (anterior) insula. We chose a whole-brain voxel-wise analysis for spatial resolution to distinguish between different areas of the orbital, medial, and lateral prefrontal cortices.

## Methods

### Study cohort and phenotyping

For this study, we analysed data from a total of 103 psychiatrically healthy subjects (53 female, 50 male) recruited from the local community. All participants gave written informed consent to study participation as part of a study protocol approved by the local ethic committee of the Medical School of Friedrich-Schiller-University of Jena, in accordance with the Declaration of Helsinki in its current version. Inclusion criteria were age 18–65 years and ability to provide informed consent, while exclusion criteria were any concurrent or previous psychiatric disorder (including current substance dependence) central nervous neurological disorders (including traumatic brain injury/loss of consciousness), or learning disability/IQ lower than 80, as well as intake of psychotropic medication.

Subjects were screened for absence of exclusion criteria, in particular any previous treatment for psychiatric disorders. IQ was estimated using the MWT-B (Mehrfachwortschatztest B;^36,37^), and while IQ scores lower 70 would be considered suggestive of a learning disability, we defined an exclusion threshold of 80 to take into account imprecisions and potential overestimations of this screening test (ultimately, however, none of our recruited subjects was excluded as the minimum detected IQ in this sample was 88). Following screening and formal inclusion, subjects underwent MRI scanning and phenotyping for narcissistic traits.

We used the narcissistic personality inventory NPI^11^, applying the full 40-item validated German version^38^, to characterise our sample for narcissistic traits. The NPI has been used in a large number of studies^20,39^, including non-clinical and clinical samples, as well as several of the functional imaging studies cited above. While validity studies of the NPI by Raskin and Terry suggested seven components defined as authority, exhibitionism, superiority, vanity, exploitativeness, entitlement, and self-sufficiency^40^, there have been alternative accounts of four factors labelled exploitativeness/entitlement, leadership/authority, superiority/arrogance, self-absorption/self-admiration^15^, and more recently of two or three factors assigned ‘power’, ‘exhibitionism’, and ‘special person’^41^. In particular, Ackerman and colleagues in a recent re-appraisal of the NPI including analyses of large college student samples^12^, advocated a three-factor model (with facets: leadership/authority, grandiose exhibitionism, and entitlement/exploitativeness). Based on findings of the validation study and factorial analysis of the German NPI translation^38^, we calculated additional seven NPI subscales designated (sample items in brackets refer to the original NPI text in English): authority (8 items, e.g.: “I am a born leader”), entitlement (6 items, e.g.; “I insist upon the respect that is due me.”), exhibitionism (7 items, e.g.: “Modesty doesn’t become me.”), exploitativeness (6 items, e.g.: “I can make anybody believe anything I want them to”), self-sufficiency (6 items, e.g.: “I rarely depend on anyone else to get things done.”), superiority (5 items, e.g.: “I think I am a special person.”), vanity (3 items, e.g.: “I like to look at myself in the mirror”).

Demographic and psychometric data of the sample are summarised in Table 1.

**Table 1:** Demographic and psychometric data of the study cohort (n = 103), including data on female (n = 53) versus male (n = 50) subjects.

	Mean (standard deviation) total sample (n = 103)	Mean (standard deviation) female subjects (n = 53)	Mean (standard deviation) male subjects (n = 50)
Age	31.7 years (10.2)	33.4 yrs (10.8)	29.9 years (9.3)
Estimated IQ (MWT-B score)	108.5 (12.5)	108.68 (12.3)	108.34 (12.8)
NPI total score	12.23 (5.73)	11.28 (5.5)	13.24 (5.9)
NPI subscale authority	3.31 (1.88)	2.96 (1.7)	3.68 (2)
NPI subscale entitlement	1.17 (1.22)	0.85 (0.9)	1.52 (1.4)
NPI subscale exhibitionism	2.01 (1.54)	1.87 (1.5)	2.16 (1.6)
NPI subscale exploitativeness	1.60 (1.22)	1.49 (1.1)	1.72 (1.3)
NPI subscale self-sufficiency	1.55 (1.28)	1.58 (1.3)	1.52 (1.3)
NPI subscale superiority	1.42 (1.03)	1.3 (1)	1.54 (1.1)
NPI subscale vanity	1.17 (1.09)	1.23 (1.1)	1.1 (1.1)

### Magnetic resonance image (MRI) acquisition

MRI scanning was done on a 3 Tesla Siemens Tim Trio system (Siemens, Erlangen, Germany) using a T1-weighted high-resolution MPRAGE sequence (magnetisation-prepared rapid gradient echo) with a standard quadrature head coil (scanning parameters: TR 2300 ms, TE 3.03 ms, flip angle α 9°, in-plane field-of-view 256 mm) acquiring 192 contiguous sagittal slices covering the whole brain. Scanning duration was 5:21 min. All scans were visually inspected after scanning for gross artefacts (e.g. movement, ghosting), and all scans passed this initial step of quality assurance.

### Voxel-based morphometry

We used a voxel-based morphometry (VBM) approach to analyse T1 scans, using Statistical Parametric Mapping (SPM) software (Wellcome Institute of Imaging Neuroscience, Institute of Neurology, London, UK) running on Matlab (Mathworks, Natik, MA, USA) and the VBM8 toolbox, r435 (C. Gaser, Jena University Hospital; http://www.dbm.neuro.uni-jena.de/vbm/vbm8), as in two previous studies of personality traits and narcissistic personality disorder, respectively^42,43^. Our processing pipeline have been described previously (e.g.^43^), including augmentation of segmentation through accounting for partial volume effects^44^, adaptive maximum a posteriori estimation^45^, and hidden Markov Random Field models^46^. All scans passed the automated quality assurance protocol in VBM8. After segmentation of grey matter maps, we applied an internal grey matter threshold of 0.2, in order to eliminate potential artefacts at ambiguous grey matter borders; this threshold is more conservative than the often used 0.1 GM threshold. Anatomical labelling was available with the AAL atlas^47^.

### Statistical analysis

For all VBM statistical analysis, we used threshold-free cluster enhancement (TFCE), an approach introduced to increase sensitivity of voxel-based analyses^48,49^, applying 5000 permutations (Smith method).

First, we tested our main hypothesis of brain structural associations with NPI scores using a general linear model (GLM) in SPM with NPI total score as regressor and age and sex as nuisance variables (in order to remove age and sex related effects). Based on TFCE, we then used a *p* = 0.05 family-wise error (FWE) correction to correct for multiple comparisons across whole-brain GM voxels, testing for both positive and negative correlations. NPI skewness of 0.411 was in an acceptable range for this statistical approach.

Second, we followed up our main analysis by testing the hypothesis of sex interactions, i.e. that correlation slopes might differ significantly between female and male study participants. For this purpose, we set up a new GLM, again using age as a regressor, to reveal areas in which female subjects would show a higher/steeper increase over males and vice versa. This analysis aimed at replicating the previous finding^35^ of sexually dimorphic associations for the parietal cortex in a VBM study (with unclear main effects of NPI total scores).

The exploratory nature of this analysis acknowledges limited statistical power in these (smaller) subgroups of the study cohort, as well as interaction effects in VBM often being more difficult to detect given lack of sensitivity even in decent sized samples.

Third, we performed exploratory analyses testing for potential associations of the seven NPI subscales with brain structure, defining separate GLMs, each including the respective NPI subscale, as well as age and sex as nuisance variables.

## Results

### Associations of NPI total score with brain structure

**Figure 1:**
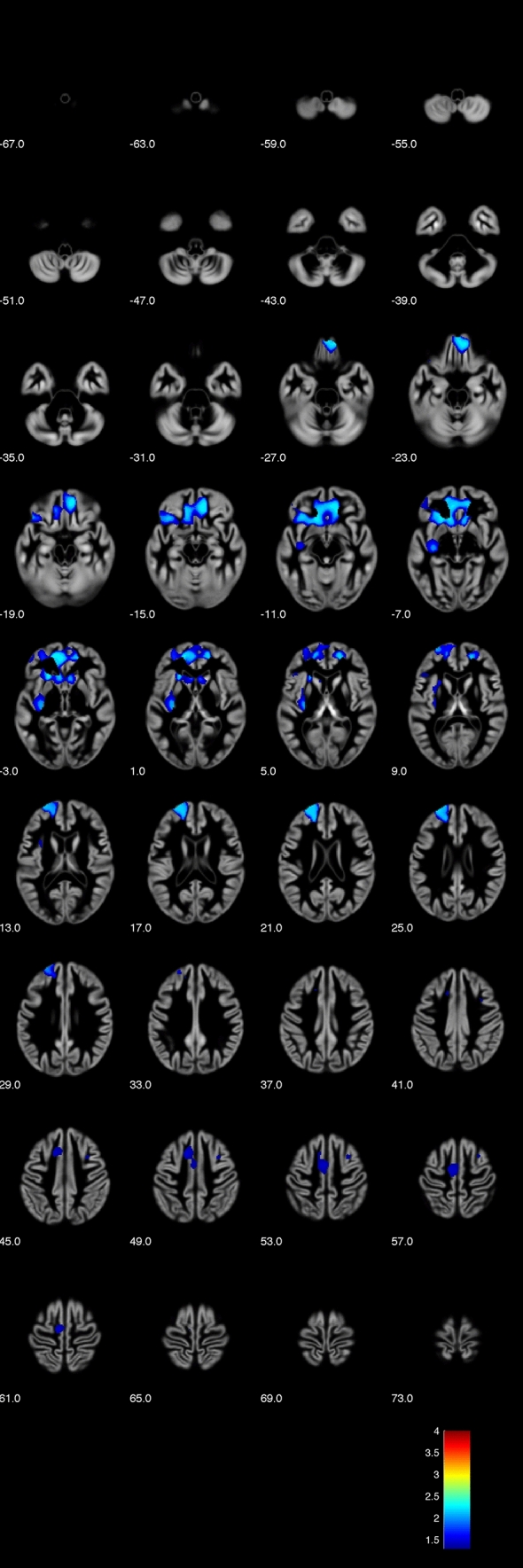
Voxel-based morphometry (VBM) analysis showing positive correlations of narcissistic personality inventory (NPI) total score with grey matter (TFCE analysis, *p* < 0.05 FWE corrected, axial sections with z levels given beneath each section) (Image created using the VBM8 toolbox, version r435; C. Gaser, Structural Brain Mapping Group, Jena University Hospital, Jena, Germany; http://www.dbm.neuro.uni-jena.de/vbm/vbm8).

**Figure 2:**
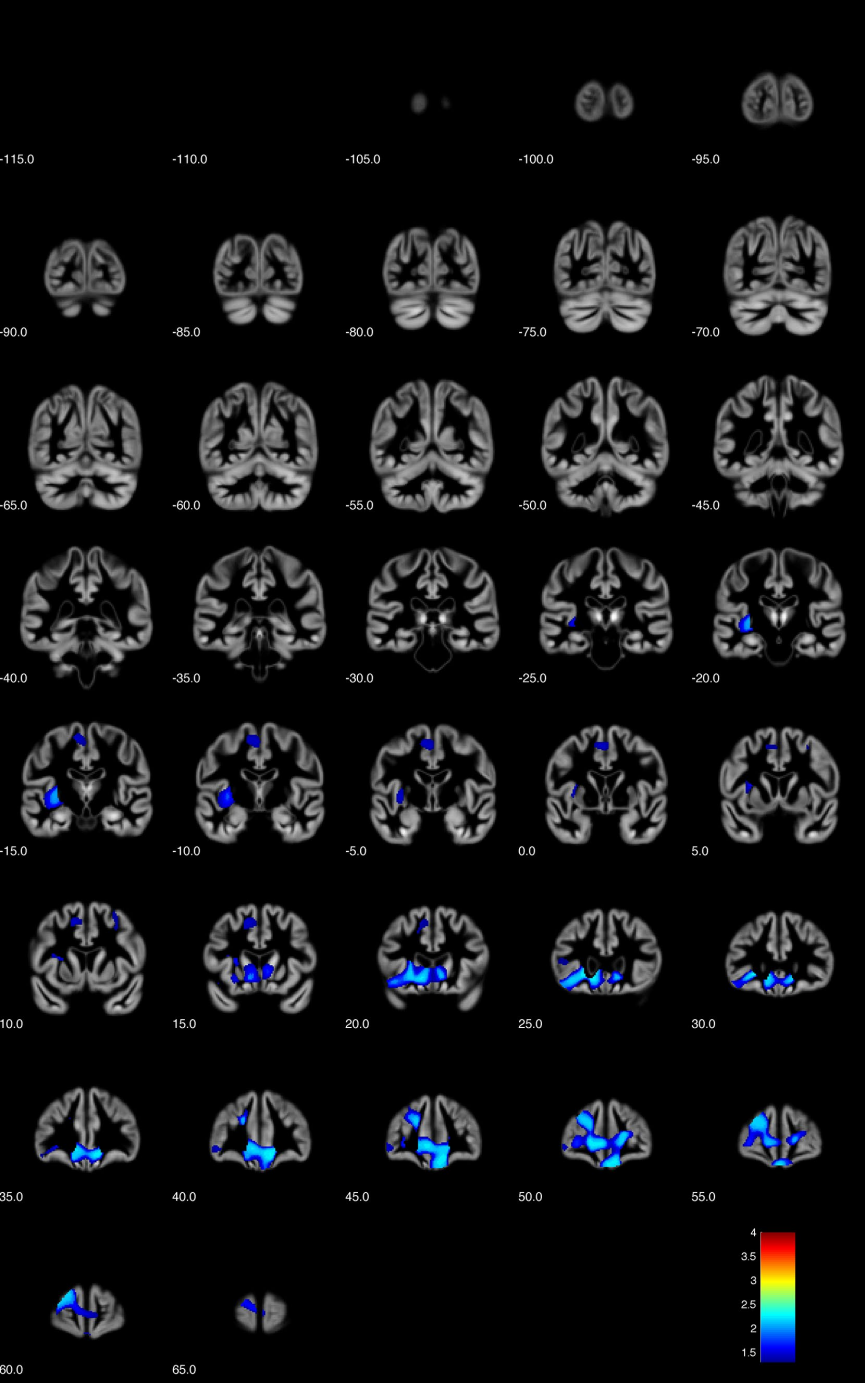
Voxel-based morphometry (VBM) analysis showing positive correlations of narcissistic personality inventory (NPI) total score with grey matter (TFCE analysis, *p* < 0.05 FWE corrected, coronal sections with y levels given beneath each section) (Image created using the VBM8 toolbox, version r435; C. Gaser, Structural Brain Mapping Group, Jena University Hospital, Jena, Germany; http://www.dbm.neuro.uni-jena.de/vbm/vbm8).

In our main analysis, we found significant (*p* < 0.05, FWE-corrected, TFCE) positive correlations NPI total scores with regional brain grey matter volume in four clusters including bilateral medial, orbital, and dorsolateral prefrontal as well as left insular cortices (see Figs. 1 and 2).

Of the four significant clusters, the first cluster spanned a large confluence of regions mostly covering the prefrontal areas (cluster size k = 15,419, maximum intensity voxel at MNI space co-ordinates 10; 39; − 14 with p_FWE-corr_ = 0.005, with additional local maxima at 10; 47; − 20 and − 21; 24; 6 – both at p_FWE-corr_ = 0.006).

Additional clusters were k = 1377 voxels (maximum intensity voxel at − 12; 18; 48 with p_FWE-corr_ = 0.035; additional local maxima − 9; − 15; 60 with p_FWE-corr_ = 0.036 and − 3; − 4; 52 with p_FWE-corr_ = 0.037), and smaller clusters with k = 178 voxels (maximum intensity voxel at − 30; 9; 40 with p_FWE-corr_ = 0.045; additional local maxima 32;8;49 with p_FWE-corr_ = 0.047 and − 30; 11; 57 with p_FWE-corr_ = 0.047) and k = 102 voxels (maximum intensity voxel at − 44; 27; 7 with p_FWE-corr_ = 0.047), respectively, with clusters extending towards bilateral caudate nuclei.

### Interaction effects with NPI total scores

We did not identify a significant interaction effect of sex and NPI total scores on brain structure at corrected thresholds (*p* < 0.05, FWE-corrected, TFCE) apart from one single voxel in the right lateral prefrontal cortex (k = 1; 58; 22; 3, p_FWE-corr_ = 0.05) with higher correlation slopes in women. In particular, we did not identify any sex-by-NPI interaction in the superior parietal cortex, as implicated in a previous study^35^.

In further exploratory analysis at uncorrected threshold levels (*p* < 0.001, uncorr.), women showed steeper positive correlations with NPI total scores than men in two right dorsolateral prefrontal clusters (k = 930; maximum intensity voxel 58; 22; 3; and k = 233; 36;26;28) and one in the right posterior parietal/occipital cortex (k = 116; 30; − 81; 40) and one single voxel at 8; − 25; 73. There were no inverse effects (i.e. steeper slopes in men compared to women) even at *p* < 0.001 uncorrected thresholds.

Comparison of psychometric data between female and male participants did not show significant group-level differences, apart from one single scale with male subjects scoring higher on the NPI subscale entitlement (T-test: T = 2.898, *p* = 0.005; assuming unequal variances based on Levene-test F = 11.154, *p* = 0.001), and trend-level findings for higher values of total NPI score in male subjects (T-test: T = 1.749, *p* = 0.083; assuming equal variance based on Levene-text F = 0.19, *p* = 0.664), and higher values for NPI subscale authority in male subjects (T-test: T = 1.956, *p* = 0.053; assuming unequal variances based on Levene-test F = 4.216, *p* = 0.043).

### Exploratory analysis of brain structure and NPI subscales

Exploratory analysis of the seven NPI subscales (authority, entitlement, exhibitionism, exploitativeness, self-sufficiency, superiority, vanity) revealed only small minor clusters in the following associations (only those with k > 15 reported): (a) for exhibitionism a positive correlation with two clusters in the left parietal lobe (k = 124; maximum at – 36; − 40; 52 with p_FWE-corr_ = 0.047) and right medial parietal/cingulate cortex (k = 17; maximum at 12; − 28; 33 with p_FWE-corr_ = 0.048), (b) for self-sufficiency a positive correlation with a cluster in the left medial prefrontal cortex (k = 84; maximum at – 10; 12; − 11 with p_FWE-corr_ = 0.048), (c) for superiority a positive correlation with a left anterior/rostral prefrontal cluster (k = 308; maximum at − 21; 56; 21 with p_FWE-corr_ = 0.032). However, we did not identify any other significant association on the brain structural level at p_FWE-corr_ < 0.05 levels. While this exploratory analysis initially used uncorrected *p* < 0.001 thresholds, it is noteworthy that none of the above clusters would survive Bonferroni adjustment for multiple comparisons (across multiple GLMs).

## Discussion

The present study set out to test the hypothesis that subclinical narcissistic traits in a nonclinical population would be associated with brain structural variation of grey matter, esp. in prefrontal systems. And indeed, our findings provide evidence of a correlation of prefrontal cortical grey matter with NPI narcissism. Our interpretation of results is directed at the three main aspects of the study: first, the implication of insular and prefrontal cortical regions (including orbitofrontal, ventromedial/medial prefrontal, and dorsolateral prefrontal areas) towards a neurobiological model of narcissistic traits; second, the relation of our findings to the (limited) imaging studies in clinical narcissistic personality disorder (NaPD); and thirdly, an overlap of our findings with studies of related behavioural traits, such as social dominance or self-enhancement, which map to some of the identified regions.

Our findings extend the previous structural association studies of narcissism (measured with the PNI) and reduced right dorsolateral prefrontal thickness^34^ by showing a (positive) correlation with a more widespread network of prefrontal areas including the medial/ventromedial and orbitofrontal cortices, subgenual anterior cingular as well as insular cortices. It is therefore the first to suggest multiple widespread prefrontal networks to be involved in the narcissistic phenotype. This is of relevance, esp. given a previous VBM study failing to demonstrate such an association^35^. This seems plausible, also given the multiple facets of narcissism on the phenotype level^1,50^, which do not make convergence on a single neuroanatomical region/network plausible. In fact, the insular finding potentially links our finding to both studies of cognitive empathy^27,51,52^ as well as to studies in patients with clinical narcissistic personality disorder^52^. However, the latter study, similar to another pilot study in NaPD^42^, only had small sample sizes, and rather hinted to a lateral prefrontal deficit. It is worthwhile noting that, unlike the clinical studies, our findings showed a *positive*, rather than negative, correlation of the narcissistic phenotype with brain volumes. It is interesting to note that comparable VBM studies of nonclinical population assessing subclinical phenotypes, for example irritability/hostility^53^ or impulsivity^54^ have shown such positive correlations and it has been suggested that this might be due to a non-linear association across a broader continuum (from nonclinical to pathology), of which only a small proportion would be assessed in a nonclinical study; hence, if narcissism, like irritability or hostility would show an inverted-U-shape relation across the whole nonclinical-to-clinical spectrum, a study in the lower to mid nonclinical range might show positive correlations (see, e.g.^53^). An additional interpretation might be that some aspects of narcissistic traits in a low expression, might be beneficial or even desirable in a particular (e.g. competitive) social context, but our lack of relevant social or other personality data in this sample does not allow for further testing in this particular cohort.

In comparing our findings to the literature, we also need to consider differences across narcissism inventories: in contrast to the NPI, the PNI focuses more on pathological narcissism, with a more thorough focus on vulnerable facets, which might be more closely associated with clinically relevant phenotypes (for discussion, see^3,8,55^).

The discrepancies to the two previous nonclinical association studies using the PNI^34^ and NPI^35^, respectively, might additionally be explained by data analysis methodology as well as culturally different expressions (e.g., see^56^).

While our study only assessed brain structure, there are several links to functional imaging studies pertinent to aspects of the narcissistic phenotype, which link our findings to prefrontal and insular networks to the expression of relevant behaviours. One of these is social rejection, which has been related to networks including the anterior insula, dorsal ACC and subgenual ACC^29^—part of which also featured prominently in our findings. Similarly, a recent study on cognitive emotion regulation training demonstrated that vmPFC activity exerts a modulated emotional response in regulating emotions to aversive images^57^, which connects our study to previous hypotheses of deficient emotion regulation in narcissism and prefrontal brain networks. The mPFC, also identified in our study, has previously been linked to self-enhancement in a series of brain stimulation studies^58–60^.

Given the relative paucity of imaging studies of narcissistic traits in the narrow sense, we should like to point out that several previous studies have linked medial PFC structure and activity to social functions, especially pertaining to social dominance and self-enhancement. The “dominance behavioral system”, which has been linked to narcissistic and manic temperament phenotypes^61,62^ provides such a framework. In fact, at least two recent fMRI studies of social dominance and hierarchies show brain activation foci in location similar to findings of our study: one showed social hierarchy processing in an anterior dorsolateral prefrontal cluster, slightly dorsal in localisation to our anterior prefrontal clusters^63^, while another showed modulation of dominance and subordination to a medial prefrontal/bilateral caudate network^64^. While the latter in particular are consistent with more general conceptualisations of biological dominance, it should be pointed out that this inference is indirect at best, and that this interpretation should be considered with caution. It should, however, be noted that networks involving mPFC activity have consistently been linked to socially dominant behaviours even across a more general biological conceptualisation of this phenotype across species^27,65–68^, which warrants further studies of its overlap with the narcissistic phenotype studied in our sample.

Our study only found minor interactions of sex and narcissism in its relation to brain structure. While we need to consider that our sample showed only minor differences in narcissism (sub)scales between females and males, it might lack generalisability in that respect (as gender differences have been shown in large meta-analyses^21^). The few findings of a sexually dimorphic effect were, however identified in the lateral prefrontal cortex and thus no effects or trends were observed in medial prefrontal, orbitofrontal, or insular cortices.

Finally, we need to consider a few limitations of our study, including the moderate sample size, which is also a potentially limiting factor in identifying sex interactions and correlations to those subscores, which are based on a smaller number of NPI items, as well as the lack of functional MRI analyses. While our choice of the NPI was guided based on its wide-spread application in the past, it might not cover some aspects of narcissism as well as other inventories, and further studies are needed to differentiate the contribution of, for example, entitlement vs. vulnerability to the different prefrontal network nodes. Despite our support for prefrontal involvement in narcissism, the current evidence across the few available studies is not unequivocal, and additional studies using more fine-grained phenotyping as well as possibly additional imaging modalities are needed to further corroborate the available evidence, which is non unequivocal.

One major limitation is specificity: as our phenotyping only included the NPI, which defines a complex, multi-faceted narcissism phenotype, we cannot exclude the possibility that other, less-specific factors or even traits unrelated to narcissism (e.g. neuroticism) might similarly have explained variance in the identified brain structure. Further studies with more in-depth phenotyping would be necessary to ascertain specificity and better characterise which singular facets of narcissism or related traits might drive the associations to different brain areas, esp. across the prefrontal cortex. Nevertheless, our study is a potentially important advance over previous studies, as it shows for the first time, using a robust imaging and statistical approach, that multiple prefrontal and insular cortical areas are correlated with the expression of narcissistic traits, even in the absence of manifest pathology.
